# The effect of dual-task on postural control and gait in individuals with Down syndrome: a systematic review

**DOI:** 10.3389/fneur.2026.1858461

**Published:** 2026-06-11

**Authors:** Gülsüm Kargı, Kimia Karimi, Parisa Sedaghati, Mohammad Alghosi, Mohammad Alimoradi, Andreas Konrad, Ramila Abedi Azar

**Affiliations:** 1 Department of Recreation Management, Faculty of Tourism, Selcuk University, Konya, Türkiye; 2 Department of Sports Injury and Corrective Exercise, Faculty of Physical Education and Sport Sciences, University of Guilan, Rasht, Iran; 3 Department of Physical Education, Technical and Vocational University (TVU), Tehran, Iran; 4 Department of Sports Injuries and Corrective Exercises, Faculty of Sports Sciences, Shahid Bahonar University of Kerman, Kerman, Iran; 5 HERC-Health, Exercise & Research Center, Dubai, United Arab Emirates; 6 Institute of Human Movement Science, Sport and Health, Graz University, Graz, Austria; 7 Laboratory for Robotic Research, Iran University of Science and Technology, Tehran, Iran

**Keywords:** balance, cognitive-motor interference, Down syndrome, dual-task, gait, postural control

## Abstract

**Background and aims:**

Individuals with Down syndrome (DS) exhibit deficits in postural control (PC) and gait. Dual-task (DT) paradigms, which involve performing concurrent cognitive or motor tasks, may exacerbate these deficits or, conversely, serve as effective interventions.

**Objective:**

This systematic review synthesizes evidence on how DT conditions affect PC and gait in individuals with DS, examining both acute effects and long-term training outcomes.

**Methods:**

PubMed, Web of Science, and Scopus were searched from inception to February 2025. Included studies examined DT effects on PC or gait in DS populations. Risk of bias was assessed using ROBINS-I (non-randomized studies; non-RCTs) and RoB-2 (randomized controlled trials; RCTs). Due to heterogeneity in the outcome measures, a narrative synthesis following SWiM guidelines was conducted.

**Results:**

Ten studies [363 participants; mean age 13.66 ± 2.53 years; eight non-RCTs (including six with control groups) and two RCTs] met inclusion criteria. Eight studies examining acute DT effects demonstrated that concurrent cognitive or motor tasks significantly impaired gait parameters (reduced velocity, increased step time, prolonged double support phase) and increased postural sway in individuals with DS compared to single-task conditions. These deficits were observed across various DT paradigms, including counting, word generation, and object manipulation. Conversely, two long-term DT intervention studies (8 weeks) reported significant improvements in dynamic balance, functional independence, and DT performance. IQ scores, reported in six studies (mean range: 26.97–66.60), correlated positively with gait speed and step length. Risk of bias was moderate in seven studies, low in two, and raised some concerns in one.

**Conclusion:**

Acute DT conditions compromise PC and gait in individuals with DS, reflecting attention resource limitations. However, preliminary evidence suggests that DT training may improve long-term functional outcomes. Longitudinal, high-quality RCTs with standardized protocols and comprehensive cognitive assessment are urgently needed to establish evidence-based DT interventions for this population and determine whether initial improvements are sustained over time.

**Systematic review registration:**

https://www.crd.york.ac.uk/PROSPERO/view/CRD42023483849, CRD42023483849.

## 1 Introduction

Down syndrome (DS) is a prevalent genetic condition linked to neurodevelopmental challenges (1). The estimated global incidence of DS is 1–10 per 1,000 live births worldwide, according to the World Health Organization (2). Individuals with DS often experience uncoordinated, slower, and variable movements, which can lead to hesitancy in their actions (3). This lack of coordination and speed can further limit their ability to respond effectively to environmental changes (3). Postural control (PC) refers to the ability to manage and maintain the body posture and position in space (4). This ability is influenced by the interaction between various sensory systems (somatosensory, visual, and vestibular) and the motor system (5). The vestibulocerebellar system controls movement, posture, and balance, and deficiencies in this system can affect motor skills and posture in individuals with DS (3, 6). Neuromuscular anomalies such as hypotonia and muscle weakness, as well as intellectual disability and sensory integration disorders, cause balance problems in individuals with DS (7). Compared to typically developing individuals, individuals with DS exhibit impairments in certain gait parameters, including greater gait variability (e.g., between-step length differences) (8, 9), and these impairments become more pronounced as task difficulty increases (8–11). Of note, this variability may help individuals with DS maintain stability by introducing flexibility into their movement patterns, thereby reducing the risk of instability or falls (12). This aligns with the idea that while gait control is generally impaired in this population, the extent of impairment can differ among individuals based on their level of functional development (13).

Dual-task (DT) examines the simultaneous performance of a postural task and a cognitive or motor task to understand the interaction and the secondary task's impact on the primary task (14). This can lead to DT interference due to the attention conflict that arises when managing both tasks simultaneously (15). The DT paradigm is commonly used to investigate attentional demands on PC (16, 17), and it has also been proposed as a valid method for exploring the interaction between cognitive and motor domains in individuals with neuromotor disorders, such as DS (18, 19). Brain structural abnormalities in individuals with DS, such as smaller frontal, occipital, and temporal lobes, reduced hippocampal volume, and decreased corpus callosum and cerebellum size, can affect cognitive processing and the planning and execution of voluntary motor actions (20). These structural changes may alter mechanisms underlying DT regulation, leading to higher DT costs, especially in children with DS compared with typical children (21, 22). Research has been conducted on the impact of cognitive demands on physical performance, like PC, across various populations, including individuals with traumatic brain injuries (23), Alzheimer’s disease (24), strokes (25), Parkinson’s disease (26), and healthy subjects (27, 28).

Although there have been some studies on the effects of DTs on PC in people with DS (29–31), to our knowledge, no systematic review has been conducted to date on the effects of DT conditions on PC in individuals with DS. Such a review would provide a comprehensive synthesis of existing evidence, highlight the limitations of previous research, and identify gaps that warrant further investigation, thereby guiding future research directions and opportunities. Many experimental studies are constrained by small sample sizes, diverse methodologies, and inconsistent findings, which limit their generalizability. For instance, several studies have relatively small sample sizes (10, 30, 31), reducing statistical power and the ability to draw broad conclusions. Additionally, the methodologies vary widely, including different DT conditions, ranging from cognitive tasks (e.g., counting, word generation) to motor tasks (e.g., carrying an object), and diverse assessment tools such as the GAITRite walkway, Timed Up and Go test, and Stabilometric Platform (32, 33). These variations make direct comparisons between studies challenging. Moreover, while some studies report significant impairments in gait and PC under DT conditions (31, 32), others report mixed results, including non-significant differences in specific gait phases (10). These inconsistencies underscore the need for a systematic review to synthesize findings and provide a clearer understanding of how DT conditions affect PC and gait in individuals with DS. A systematic review not only addresses the variability and heterogeneity in participant demographics, intervention protocols, and outcome measures across studies but also highlights areas where future research is needed. Therefore, this systematic review aims to systematically aggregate studies that assessed the effects of DT on PC and gait in individuals with DS.

## 2 Methods

### 2.1 Protocol and registration

The systematic review followed the Preferred Reporting Items for Systematic Reviews and Meta-Analyses (PRISMA) guidelines (34) and was registered in the International Prospective Register of Systematic Reviews (PROSPERO) under registration number CRD42023483849.

### 2.2 Eligibility criteria

After the search phase, two authors, acting independently, meticulously reviewed all titles and abstracts generated by the search strategy. The systematic review’s inclusion of studies was meticulously guided by the Population, Intervention, Comparison, Outcome, and Study Design (PICOS) framework, ensuring a comprehensive and structured approach to study selection (Table 1).

**Table 1:** Selection criteria for studies.

Inclusion criteria		Exclusion criteria
Population	Individuals with Down syndrome, of any sex or age.	History of lower extremity or spine surgery; non-human studies.
Intervention	*For acute dual-task studies*: Single-session performance of a secondary task (cognitive or motor) concurrently with a postural or gait task. *For dual-task training studies*: Multi-session dual-task training program (any duration).	Multicomponent interventions (i.e., precluding the study of the isolated effect of dual-tasks).
Comparison	*For acute dual-task studies*: Single-task condition (postural or gait task performed alone) as within-subject comparator. *For dual-task training studies*: Passive control group (no intervention), active control group (single-task training, placebo/sham, or alternative non-DT intervention), or within-subject pre-post comparison.	Not applicable.
Outcome	Postural control was assessed with validated measures (e.g., center of pressure, center of gravity, center of mass, functional scales).	Studies that did not measure postural control or gait outcomes.
	Gait variables (e.g., step length, step width, stride length, stride width, single-leg support time, double-leg support time, step time, gait velocity, gait speed, heel strike time, foot flat time, mid-stance time, heel-off time, toe-off time, walking time, walking speed, number of steps, cadence, gait deviation index).	
Study design	Non-randomized controlled trials; randomized controlled trials; single-group studies with within-subject comparisons.	Studies without subject comparisons; reviews.

### 2.3 Search strategy

The search included the electronic databases Web of Science, PubMed, and Scopus, from inception to February 25th, 2025, with two authors (PS and MALG) searching independently; discrepancies were resolved through discussion and, if needed, the opinion of a third author (MALI). The search strategy used specific MeSH terms (for PubMed) and text words/phrases, combined using Boolean operators [e.g., (“dual task” OR “dual-task*” OR “divided attention” OR “concurrent task” OR “cognitive task” OR “multi task” OR “double task” OR “motor task” OR “cognitive-motor interference” OR “secondary task” OR “walking while talking”) AND (posture OR “postural control” OR “postural sway” OR “postural stability” OR “postural steadiness” OR balance OR equilibrium OR “postural balance” OR mobility OR ambulation) AND (“down syndrome” OR “down’s syndrome” OR “21 trisomy” OR “trisomy 21”)]. The complete search strategy for each database is presented in Supplementary Table 1. Grey literature was searched via clinical trial registries (clinicaltrials.gov/, WHO ICTRP), preprint servers (medRxiv, bioRxiv), ProQuest Dissertations & Theses, conference proceedings (International Conference on Down Syndrome, World Congress of Physical Therapy, ISPGR), the first 100 Google Scholar results, and reference lists. Corresponding authors were contacted (up to 3 attempts over 4 weeks) regarding missing data or unpublished trials. No language restrictions were applied (Google Translate was used), and the retrieved studies were organized in EndNote with duplicates removed. The Connected Papers website^1^ was used to enhance the search for relevant research.

### 2.4 Data extraction

Two authors collected information from retrieved papers (GK and RAA), including study details (author, year of publication, location), study design, participant demographics [sample size, sex, age, and intelligence quotient (IQ)], DT characteristics for the intervention group, PC measures, assessment tool, main outcomes, conclusion, and risk of bias (ROBINS-I and RoB-2). If any important information was missing, the corresponding authors were contacted via email, with a maximum of 3 attempts over a 4-week period (at least 1 week between each attempt) to obtain the necessary details.

### 2.5 Quality assessment

The Cochrane guidelines were followed to assess the risk of bias in individual studies. For this systematic review, the ROBINS-I (35) tool was used for non-randomized studies (non-RCTs) and RoB-2 (36) for randomized controlled trials (RCTs). The quality of the included studies was evaluated by two authors (KK and MALG) who independently assessed the seven domains of ROBINS-I and the five domains of RoB-2 (e.g., confounding, participant selection, intervention classification, deviations from intended interventions, missing data, outcome measurement, reported result selection). For RCTs, bias arising from the randomization process was also evaluated. The authors used tools that provided detailed guidance and signaling questions to make judgments, with options such as “Low risk of bias,” “High risk of bias,” “Some concerns”/"Moderate,” and “Unclear risk of bias”/"No information,” along with supporting observations. To assess inter-rater reliability, Cohen’s kappa (*κ*) was calculated at the domain level—that is, the two authors’ independent initial judgments for each domain of each study were compared. Across all 7 domains of ROBINS-I (8 non-RCTs) and all 5 domains of RoB-2 (2 RCTs), the authors demonstrated perfect agreement (*κ* = 1.0). Any initial discrepancies were resolved through discussion and, when necessary, consultation with a third author (MALI) prior to finalizing the risk-of-bias judgments, ensuring the final dataset reflected consensus. To visually represent the risk of bias in the included studies, the authors used the Robvis tool^2^ to generate traffic light plots, as recommended by Cochrane.

### 2.6 Data synthesis

The study used narrative data synthesis and followed the PRISMA guidelines (34) to ensure thorough and transparent reporting, improving the validity of the results. Due to the heterogeneity of the outcome measures, a meta-analysis was not possible, so the review used a Synthesis Without Meta-Analysis (SWiM) approach, following the SWiM reporting guideline (37). Studies were grouped by design (acute DT studies vs. DT training studies) and outcome type (gait parameters vs. PC parameters). Direction of effect was defined as impairment in DT relative to single-task conditions in acute studies and improvement post-intervention relative to baseline or control groups in training studies.

## 3 Results

### 3.1 Study identification

**Figure 1:**
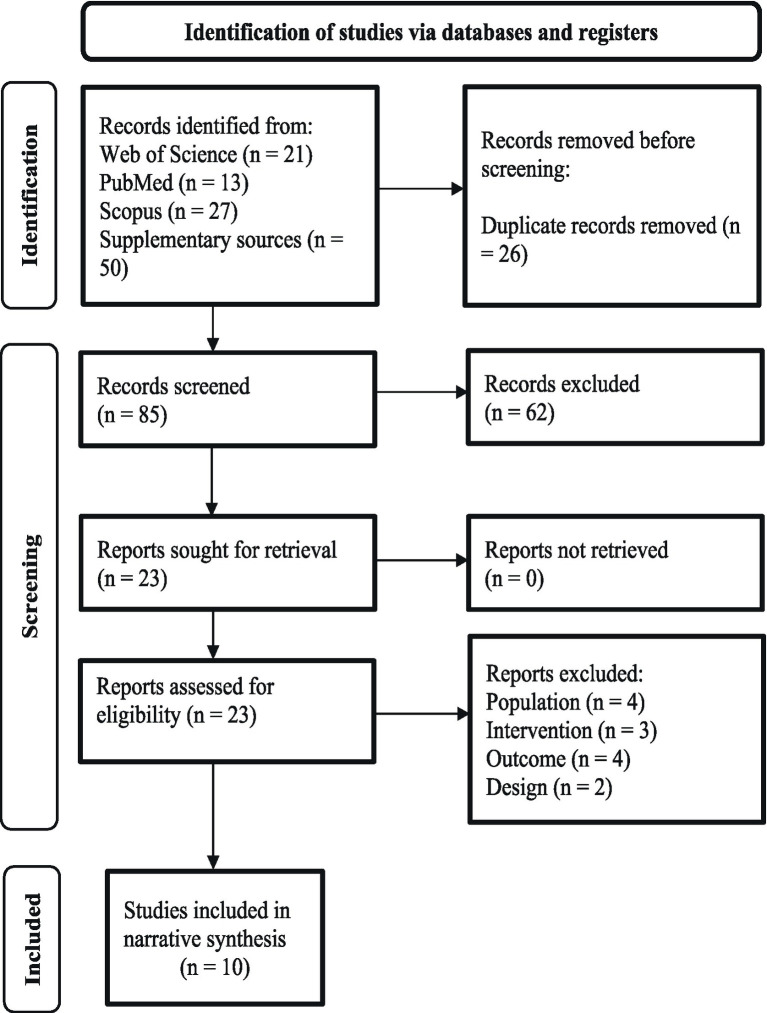
PRISMA 2020 flow diagram showing the identification, screening, eligibility, and inclusion process. Of 109 initial records, 10 studies met the inclusion criteria.

Based on the PRISMA guidelines (34), the electronic database search initially retrieved 111 articles (50 from Supplementary material). After removing duplicates, 85 studies remained for further screening based on the inclusion and exclusion criteria. The review focused on studies that investigated the effects of DT and DT exercise on individuals with DS. A summary of the study progression and the rationale for exclusions at each phase is depicted in a PRISMA diagram (Figure 1). Excluded studies are listed in the Supplementary Table 2.

### 3.2 Descriptive characteristics of the included studies

The studies were published between 2013 and 2025, and a total of 363 individuals participated. Six studies included both male and female participants (11, 18, 29, 30, 33, 38), while only one study focused exclusively on males (10). The mean age of the participants was 13.66 ± 2.53 years, and the studies examined outcomes including PC, CoP mean velocity, static and dynamic balance, and gait parameters. The average intellectual quotient score across four studies was 45.75 ± 5.27 (11, 18, 30, 32), while two studies reported a range of 35–70 (10, 38). Four studies did not provide IQ score data (29, 31, 33, 39). Of the total number of studies, eight were non-RCTs (10, 11, 18, 29, 31–33, 39), while two were RCTs (30, 38). Regarding study design, eight studies investigated acute DT effects (single session) (10, 11, 18, 29, 31–33, 39), while two studies evaluated long-term DT training interventions (8 weeks) (30, 38). Table 2 provides a summary of the specific characteristics and retrieved data of all ten studies.

**Table 2:** Characteristics of the included studies.

Study details	Design of study	Participant demographics	Dual-task	Postural control or gait measures	Assessment tool	Main outcomes	Risk of bias
Horvat et al. (31), USA	Non-RCT	*N*: 24; sex: NA; age: 22.62 ± 3.12 years; groups: DS (12) and CG (12); IQ score: NA	Carrying a tray with five cups, holding a plate in one hand and a cup in the other, buttoning a shirt, and talking on a cell phone.	Step length; step width; stride length; Stride width; single leg support time; double leg support time; step time; gait velocity.	GaitRite electronic walkway.	Spatial components showed significant group and condition interactions (*p* ≤ 0.01). Temporal components also yielded significance (*p* ≤ 0.01).	Moderate
Hocking et al. (33), Australia	Non-RCT	*N*: 52; sex: M = 26; F = 26; age: 24.33 ± 5.47 years; groups: DS (17), WS (18), and CG (17); IQ score: NA	Generating words from a specific semantic category.	Gait speed; Step time; Step length; Step width; double support time.	GAITRite walkway.	Pairwise comparisons revealed that the DS group were significantly slower when compared to the CG (*p* = 0.016), and spent a greater time in the double support phase of the gait cycle relative to controls.	Moderate
Pena et al. (29), Brazil	Non-RCT	*N*: 47; sex: M = 20; F = 27; age: 10.24 ± 2.36 years; groups: DS (21) and CG (26); IQ score: NA	DT-Bim: sit-to-stand while carrying a tray using both hands.	Postural sway.	Sit-to-stand.	Children with DS exhibited greater sway than typical children in all sit-to-stand phases (*p* < 0.001).	Moderate
			DT-Uni-Dom: sit-to-stand while holding a plastic cup simulating water using the dominant hand.				
			DT-Uni-Non-Dom: sit-to-stand movement while holding a plastic cup simulating water.				
Ghadiri et al. (10), Iran	Non-RCT	*N*: 20; sex: M = 20; age: 11.50 ± 2.28 years; groups: DS (20); IQ score: 50–70 ^ǂ^	Countdown from number 20 to the end of the route.	Heel strike time; foot flat time; mid-stance time; heel-off time; Toe-off time.	Foot medisense.	No significant difference between right and left heel strike phases (*p* > 0.05), but significant differences in other phases (*p* < 0.05).	Moderate
			Countdown by two numbers from 30 to the end of the route.				
Klotzbier et al. (18), Germany	Non-RCT	*N*: 36; sex: M = 18; F = 18; age: 8.90 ± 1.12 years; groups: DS (12), TD-CA (12), and TD-MA (12); IQ score: 66.60 ± 2.13^c^	Cognitive activity: increasing sequential numbers and increasing sequential numbers and letters.	Walking time; gait speed.	Trail-walking-test.	There were significant decreases in speed with increasing cognitive demands.	Low
Uysal et al. (39), Turkey	Non-RCT	*N*: 32; sex: NA; Age: 10.72 ± 2.94 years; Groups: DS (32); IQ score: NA	Motor activity: carrying a closed bottle filled with water.	Walking speed; number of steps.	10-meter walking test.	Children with DS showed decreased walking speed and increased number of steps during cognitive and motor dual task compared to single motor task (*p* < 0.05).	Moderate
			Cognitive-motor activity: counting consecutive numbers from 1 to 20.				
Büyükçelik et al. (30), Turkey	RCT	*N*: 27; sex: M = 18; F = 9; age: 11.44 ± 3.36 years; groups: DS as intervention group (13) and DS as control group (14); IQ score: 26.97 ± 6.25^c^	Cognitive tasks: naming colors in the room, saying names of relatives, saying names of fruits and vegetables, saying names of friends.	Dynamic balance; postural steadiness; static balance; functional balance.	Timed Up and Go; single leg stance; tandem-stance; 30 s sit to stand; pediatric balance scale.	The combined balance and strength training led to significant improvements in both balance and DT performance in the IG (*p* < 0.05).	Low
			Motor tasks: carrying an empty box, arms flexed to 90°, arms abducted to 90°.				
Borji et al. (32), Tunisia	Non-RCT	*N*: 28; sex: NA; age: 14.47 ± 1.20 years; groups: DS (15) and CG (13); IQ score: 48.00 ± 2.12^c^	Selective span task; verbal fluency task for category.	CoP mean velocity.	Static Stabilometric Platform.	Postural performance was significantly altered in the DS group during all DT conditions compared to the ST situation (*p* < 0.001).	Moderate
Ito et al. (11), Japan	Non-RCT	*N*: 68; sex: M = 29; F = 39; age: 8.75 ± 1.00; groups: DS (17) and CG (51); IQ score: 41.50 ± 10.6^c^	Cognitive activity: movie-watching tasks.	Balance function; gait speed; step length; gait deviation index; cadence.	Single-leg standing.	Children with DS showed poorer balance function and muscle strength and lower gait quality than the control group. In the DS group, there was a significant positive correlation between gait speed, step length, and intelligence quotient.	Moderate
Triki et al. (38), Tunisia	RCT	*N*:29; sex: both^b^; age: 14–17 years; groups: DTTG (10), STTG (10) and CG (9); IQ score: 35–69^a^	Motor task: balance exercises (e.g., squats, walking on a beam, single-leg stance)	CoP mean velocity	Static Stabilometric Platform	DTT improved both postural and cognitive performances (*p* < 0.001), whereas STT improved only postural performance under ST conditions. DTTG showed significant reductions in CoPVm under DT conditions.	Some concerns
			Cognitive task: selective word recall test				

CG, comparator group; CoP, centre of pressure; CoPVm, centre of pressure mean velocity; DS, down syndrome; DT, dual-task; DT-Bim, a dual-task bimanual activity; DTT, dual task training; DTTG, dual task training group; DT-Uni-Dom, a dual-task unimanual dominant activity; DT-Uni-Non-Dom, dual-task unimanual nondominant activity; F, female; IQ, intelligence quotient for DS group; M, male; N, number; NA, not applicable; non-RCT and RCT, non-randomized and randomized controlled trial, respectively; ST, single task; STT, single task training; STTG, single task training group; TD-CA, typically developing-chronologically aged matched; TD-MA, typically developing-mental-age-matched; WS, Williams syndrome.

a Reported as range.

b Reported without the numbers of males and females.

c Reported as mean and standard deviation for DS individuals.

### 3.3 Outcome

Five studies focused solely on gait parameters (10, 18, 31, 33, 39), four studies concentrated exclusively on PC variables (29, 30, 32, 38), and one study evaluated both gait and PC (11) in individuals with DS. In two studies, only motor tasks were added to gait tasks (29, 31); five studies employed cognitive tasks in addition to gait or balance tasks (10, 11, 18, 32, 33). Additionally, three studies incorporated both cognitive and motor tasks as the second task (30, 38, 39). The gait variables examined across the included studies encompassed a wide range of parameters, such as speed, step time, single leg support time, double leg support time, heel strike time, foot flat time, mid-stance time, heel-off time, toe-off time, number of steps, step length, step width, stride length, stride width, gait deviation index, and cadence (10, 11, 18, 31, 33, 39). The studies also assessed various aspects of PC, including postural sway, dynamic balance, postural steadiness, static balance, functional balance, and overall balance function (11, 29, 30, 32, 38). The studies utilized various assessment tools to evaluate PC and gait, including the GAITRite electronic walkway, sit-to-stand tests, walking tests (e.g., 10-meter walking test, Timed Up and Go), single-leg stance, tandem stance, the 30-s sit-to-stand test, the pediatric balance scale, and static Stabilometric Platforms.

#### 3.3.1 Acute dual-task effects (single session)

Eight studies examined the immediate effects of performing a secondary task (cognitive or motor) on gait and PC in individuals with DS (10, 11, 18, 29, 31–33, 39). Across these studies, the addition of a concurrent task consistently impaired performance compared to single-task conditions. Regarding gait parameters, Horvat et al. (31) reported that carrying a tray, holding a plate, buttoning a shirt, or talking on a cell phone significantly reduced gait velocity and increased step time and double-leg support time in young adults with DS (*p* < 0.05). Similarly, Hocking et al. (33) reported that during single-task walking, individuals with DS walked more slowly than controls (105.3 ± 25.9 cm/s vs. 129.8 ± 13.9 cm/s, *p* = 0.016). During DT conditions, the DS group showed significant DT costs during the concurrent digit span task (verbal working memory), including reduced speed and increased step width (*p* < 0.01). Uysal et al. (39) reported that DT conditions impaired walking in children with DS. Walking time increased from 12.5 ± 1.3 s (single-task) to 13.6 ± 1.3 s (motor DT) and 13.5 ± 1.3 s (cognitive-motor DT) (*p* < 0.001). Cadence increased from 21.9 ± 2.2 to 23.6 ± 2.6 steps/min (motor DT) and 23.2 ± 2.8 steps/min (cognitive-motor DT) (*p* < 0.001). Ghadiri et al. (10) reported that a secondary cognitive counting task prolonged stance phase phases in children with DS, including foot flat (0.31 ± 0.17 s to 0.45 ± 0.20 s), mid-stance (0.49 ± 0.25 s to 0.67 ± 0.30 s), heel-off (0.66 ± 0.34 s to 0.91 ± 0.39 s), and toe-off (0.81 ± 0.44 s to 1.13 ± 0.49 s) (*p* < 0.05), while Klotzbier et al. (18) reported that children with DS showed progressive reductions in walking speed as cognitive task demands increased (motor-only: 42.8 s; numbers: 95.2 s; numbers-letters: 109 s; *p* < 0.001). Ito et al. (11) reported that children with DS showed poorer gait quality than controls in single-task walking (77.5 vs. 93.3, *p* < 0.001). During DT (walking while watching a movie), they showed significantly greater DT costs for gait speed (−32.1% vs. −9.7%), step length (−15.7% vs. −7.0%), and cadence (−11.4% vs. −3.1%) (*p* < 0.01), but not for gait quality. IQ was positively correlated with gait speed (*r* = 0.58) and step length (*r* = 0.57). Regarding PC, Pena et al. (29) reported that children with DS showed greater postural sway than controls during sit-to-stand (*p* < 0.001). Contrary to expectations, adding a concurrent motor task (carrying a tray or cup) decreased postural sway in children with DS (*p* < 0.05), suggesting a stiffness strategy to maintain stability. Borji et al. (32) reported that PC was significantly impaired in adolescents with DS during all DT conditions compared to single-task standing (*p* < 0.001). Motor DT costs were higher during the verbal fluency task than the selective span task (*p* < 0.001). Cognitive performance also declined under DT conditions. Ito et al. (11) also found that children with DS had significantly poorer balance function (single-leg standing time: 1.3 vs. 120.0 s, *p* < 0.001).

#### 3.3.2 Long-term dual-task training effects (intervention studies)

Two RCTs investigated the effects of multi-session DT training over 8 weeks in individuals with DS (30, 38). Both studies reported positive outcomes. Büyükçelik et al. (30) reported that 8 weeks of DT balance training (twice weekly) significantly improved dynamic balance (TUG: from 11.0 to 8.8 s), static balance (single-leg stance: from 3.9 to 10.0 s; tandem stance: from 4.7 to 16.6 s), functional balance (from 50.0 to 53.4 points), and DT performance in children with DS (mean IQ: 26.97) compared to a control group (*p* < 0.05). Triki et al. (38) compared DT training (balance + word recall) to single-task training (balance alone) and a control group in adolescents with DS (IQ: 35–69). After 8 weeks, only the DT training group showed significant reductions in postural sway under DT conditions (from 32.2 to 26.2 mm/s on the firm surface and from 38.9 to 30.5 mm/s on the foam, *p* < 0.001) and improved cognitive performance (*p* < 0.001). Single-task training improved postural performance only under single-task conditions, with no cognitive gains.

### 3.4 Quality assessment

**Figure 2:**
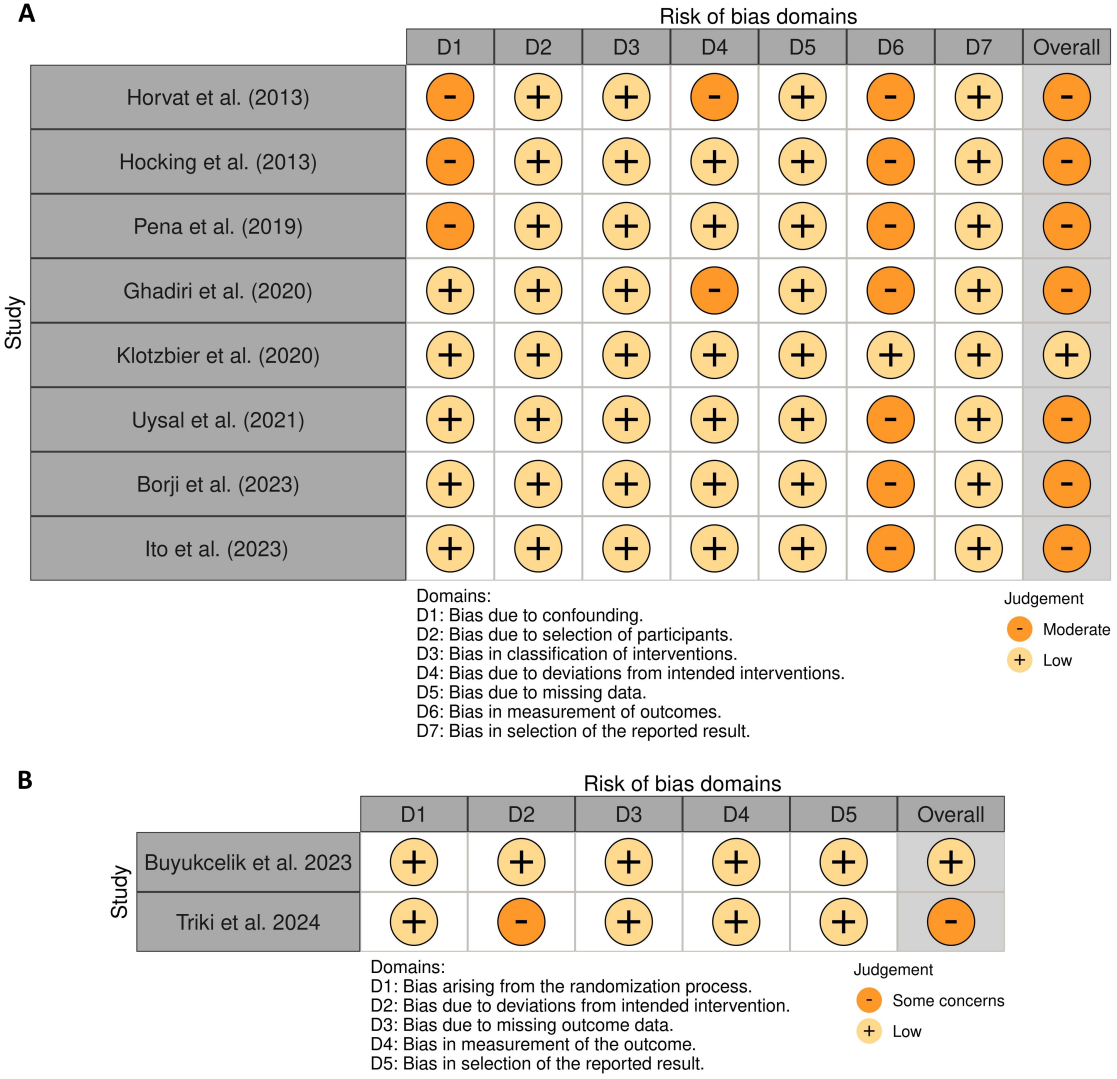
Risk of bias assessment. **(A)** ROBINS-I for non-randomized studies (*n* = 8): seven moderate, one low risk. **(B)** RoB-2 for randomized controlled trials (*n* = 2): one low risk, one with some concerns.

Risk of bias was assessed using ROBINS-I for non-RCTs (*n* = 8) and RoB-2 for RCTs (*n* = 2). Among the eight non-RCTs, seven were judged to have a moderate risk of bias (10, 11, 29, 31–33, 39), and one demonstrated a low risk of bias (18). The moderate ratings were primarily driven by confounding and by the measurement of outcomes. Conversely, all non-RCTs showed low risk for participant selection, intervention classification, missing data, and selection of reported results, as DT conditions were clearly defined, outcome data were complete, and all measured outcomes were reported (Figure 2A). For the two RCTs, Büyükçelik et al. (30) was rated as having a low risk of bias across all domains due to clear randomization, blinded outcome assessment, and complete follow-up. Triki et al. (38) raised some concerns primarily due to deviations from intended interventions, as it was unclear whether participants and personnel were blinded to group assignment during the 8-week intervention; however, other domains, including missing data, outcome measurement, and selective reporting, were low risk (Figure 2B).

## 4 Discussion

The present systematic review examined the effects of DT conditions on PC and gait in individuals with DS. The main findings indicate that individuals with DS experience significant DT interference, with acute cognitive or motor secondary tasks impairing both gait parameters and PC. However, evidence from long-term intervention studies suggests that DT training may improve balance and functional performance. These findings highlight the complex interaction between cognitive and motor processes in individuals with DS and suggest that while DT conditions initially challenge motor performance, repeated exposure may lead to beneficial adaptations.

The acute deterioration in gait and PC observed across studies may be explained by limited attentional capacity and impaired executive functioning in individuals with DS. DT paradigms require the allocation of cognitive resources to both motor and cognitive tasks simultaneously (40). However, individuals with DS often exhibit deficits in working memory, attention, and executive control, which may compromise their ability to divide attention efficiently between tasks (10, 20, 21). As a result, performance in one or both tasks deteriorates under DT conditions. Furthermore, structural and functional brain differences commonly reported in individuals with DS, including reduced cerebellar volume, smaller frontal lobes, and corpus callosum abnormalities, may contribute to impaired motor planning and coordination (20). These neurological differences may limit the ability to integrate sensory input and execute coordinated motor responses, particularly when cognitive demands increase. Consequently, individuals with DS may adopt compensatory strategies such as reducing gait speed, increasing double-support time, and widening step width to maintain stability and reduce fall risk. The findings of Horvat et al. (31), Hocking et al. (33), and Uysal et al. (39) support this interpretation, demonstrating reduced walking speed and increased step variability during DT conditions. Similarly, studies examining PC reported increased postural sway and reduced balance performance during concurrent tasks (29, 32). These adaptations may represent protective mechanisms that prioritize stability over efficiency when attentional demands exceed available cognitive resources.

In contrast to acute impairments, long-term DT training demonstrated beneficial effects on balance and functional performance. The RCTs included in this review reported improvements in dynamic balance, static balance, and functional mobility following 8 weeks of DT training (30, 38). These findings suggest that repeated exposure to DT conditions may facilitate motor learning and improve the integration of cognitive and motor processes. One possible explanation for these improvements is the development of motor automaticity. Through repeated practice, individuals may learn to perform motor tasks more automatically, thereby reducing the cognitive load associated with movement execution (41). This increased automaticity may allow individuals with DS to allocate attentional resources more effectively during DT conditions. Additionally, DT training may promote neuroplastic adaptations that enhance coordination and sensory integration (42). Therefore, preliminary evidence suggests that DT training may have potential benefits as a rehabilitation strategy for individuals with DS.

In the absence of established minimal clinically important difference (MCID) values specifically validated for individuals with DS, the practical relevance of statistically significant improvements must be interpreted cautiously. Nevertheless, several findings warrant interpretive consideration. Regarding the TUG test, the improvement from 11.0 to 8.8 s reported by Büyükçelik et al. (30) approaches functional benchmarks where times below 10 s are associated with independent mobility in other populations. Similarly, the increase in single-leg stance time from 3.9 to 10.0 s represents a transition from marked instability to near-functional durations, potentially relevant for daily activities such as dressing or stepping over obstacles. The reduction in center-of-pressure velocity reported by Triki et al. (38) (approximately 6–8 mm/s) indicates improved postural steadiness. These improvements may have meaningful implications for daily function and fall risk reduction, though DS-specific validation of MCID thresholds remains lacking. Children and adolescents with DS often exhibit delayed motor development, hypotonia, ligament laxity, and reduced muscle strength, all of which contribute to gait abnormalities and impaired PC (43). Previous studies have reported increased joint stiffness, shorter step lengths, and wider step widths in individuals with DS (44). These characteristics are often interpreted as compensatory strategies aimed at enhancing stability by increasing the base of support and reducing the risk of falls (12). However, while such adaptations may improve stability, they can also reduce gait efficiency and increase energy expenditure during walking. For example, Uysal et al. (39) reported decreased walking speed and increased step count during DT conditions, while Horvat et al. (31) observed less efficient movement patterns when individuals performed concurrent tasks. These findings suggest that individuals with DS may struggle to adapt motor strategies when cognitive demands increase. Moreover, individuals with DS may rely more on conscious control of movement than on automatic motor processes. When attention is divided during DT conditions, fewer cognitive resources remain available for motor control, resulting in decreased gait stability and altered movement patterns (18, 31, 32). This may explain the observed increase in double-support time, reduced gait speed, and increased variability reported across several studies. These adaptations may reflect a protective strategy to maintain balance, but they may also increase the risk of functional limitations in daily life, particularly in environments that require simultaneous cognitive and motor performance, such as walking while conversing or navigating obstacles.

Additionally, the variation in IQ reported across studies is another important factor influencing DT performance in individuals with DS. Lower IQ levels have been associated with greater difficulty managing postural disturbances and maintaining balance (45). The included studies reported wide variations in IQ scores, ranging from approximately 26–70, which may partially explain the heterogeneity in results. Ito et al. (11) reported a positive correlation between gait speed, step length, and IQ, suggesting that cognitive ability may influence motor performance. Individuals with higher cognitive functioning may be better able to allocate attentional resources and manage DT demands. Conversely, individuals with lower cognitive abilities may demonstrate greater DT interference. These findings highlight the importance of considering cognitive function when designing rehabilitation programs for individuals with DS.

The findings of this review have important clinical implications. Rehabilitation programs for individuals with DS should consider incorporating DT training to improve functional mobility and balance. Clinicians may begin with simple DT activities and progressively increase task complexity as individuals develop greater motor automaticity and cognitive capacity. Additionally, individualized training programs that consider cognitive ability and motor function may enhance outcomes. Despite the valuable insights this review provides, several limitations should be acknowledged. First, the number of included studies was relatively small, limiting the strength of conclusions. Second, heterogeneity in participant characteristics, assessment tools, and DT paradigms made direct comparisons challenging. Third, variations in IQ and cognitive abilities across studies may have influenced results. Fourth, sample sizes were small across most included studies, reducing statistical power and generalizability. Fifth, only two RCTs were identified, neither with follow-up assessments to determine the durability of training effects. Sixth, cognitive assessment tools varied considerably across studies, and no validated DS-specific battery for executive function was available. Seventh, no study analyzed outcomes by age subgroup despite the broad age range, spanning critical developmental periods. Eighth, IQ data were missing in four studies (29, 31, 33, 39), limiting analysis of the relationship between cognitive level and DT performance. Ninth, the absence of validated MCID values for gait and PC outcomes in individuals with DS precludes definitive conclusions about the clinical significance of observed improvements, including their translation to fall risk reduction and functional independence. Future research should include larger sample sizes and adequately powered RCTs with long-term follow-up to assess durability; use standardized DT paradigms and a validated DS-specific executive function battery; analyze outcomes by age subgroups; and systematically account for or report IQ data to clarify the relationship between cognitive level and DT performance.

## 5 Conclusion

Individuals with DS may have difficulty maintaining PC during DTs due to movement coordination challenges, neurodevelopmental characteristics, and gait abnormalities. DT exercises show promise in improving their PC and stability. However, this conclusion is based on only two long-term intervention studies. More high-quality research, particularly large-scale RCTs with adequate statistical power, is urgently needed to better understand and address the specific challenges and needs of this population.

## Data Availability

All data generated or analyzed during this study are included in this published article and Supplementary materials, further inquiries can be directed to the corresponding authors.

## Footnotes

1
https://www.connectedpapers.com/

2
www.riskofbias.info/

## References

[1] Yenturk Sisman B Celik NM Koroglu M. Comparison of motor characteristics between judokas and sedentary children with Down syndrome. JROLSS. (2025) 6:836–45. doi: 10.70736/jrolss.2107

[2] Al-Biltagi M. (Ed.) Down Syndrome Children—An Update. Sharjah: Bentham Science Publishers (2015).

[3] Horvat M Croce R Fallaize A. Information processing and motor control in Down syndrome. J Down Syndr Chr Abnorm. (2016) 2:107. doi: 10.4172/2472-1115.1000107

[4] Agmon M Lavie L Doumas M. The association between hearing loss, postural control, and mobility in older adults: a systematic review. J Am Acad Audiol. (2017) 28:575–88. doi: 10.3766/jaaa.16044, 28590900

[5] Berenshteyn Y Gibson K Hackett GC Trem AB Wilhelm M. Is standing balance altered in individuals with chronic low back pain? A systematic review. Disabil Rehabil. (2019) 41:1514–23. doi: 10.1080/09638288.2018.143324 29382241

[6] Carvalho RL Vasconcelos DA. "Motor behavior in Down syndrome: atypical sensoriomotor control". In: ed. Dey S. Prenatal Diagnosis and Screening for Down Syndrome. London. (2011). p. 33–42.

[7] Malak R Kotwicka M Krawczyk-Wasielewska A Mojs E Szamborski W. Motor skills, cognitive development and balance functions of children with Down syndrome. Ann Agric Environ Med. (2013) 20:803–6. 24364457

[8] Naito M Aoki S Kamide A Miyamura K Honda M Nagai A. Gait analysis in Down syndrome pediatric patients using a sheet-type gait analyzer: pilot study. Pediatr Int. (2015) 57:860–3. doi: 10.1111/ped.12691, 25998919

[9] Smith BA Stergiou N Ulrich BD. Patterns of gait variability across the lifespan in persons with and without Down syndrome. J Neurol Phys Ther. (2011) 35:170–7. doi: 10.1097/npt.0b013e3182386de1, 22052133 PMC3223537

[10] Ghadiri F Mosadegh Y Alghosi M Golzari Z. The effect of secondary cognitive task on time of the stance phase of people with Down syndrome. Neurosci J Shefaye Khatam. (2020) 9:36–44. doi: 10.52547/shefa.9.1.36

[11] Ito Y Ito T Ohno A Kubota T Tanemura K Narahara S. Gait performance and dual-task costs in school-aged children with Down syndrome. Brain Dev. (2023) 45:171–8. doi: 10.1016/j.braindev.2022.11.001, 36424235

[12] Zago M Duarte NAC Grecco LAC Condoluci C Oliveira CS Galli M. Gait and postural control patterns and rehabilitation in Down syndrome: a systematic review. J Phys Ther Sci. (2020) 32:303–14. doi: 10.1589/jpts.32.303, 32273655 PMC7113426

[13] Rigoldi C Galli M Albertini G. Gait development during lifespan in subjects with Down syndrome. Res Dev Disabil. (2011) 32:158–63. doi: 10.1016/j.ridd.2010.09.009, 20943345

[14] De Freitas TB Leite PHW Doná F. The effects of dual task gait and balance training in Parkinson’s disease: a systematic review. Physiother Theory Pract. (2018) 36:1088–96. doi: 10.1080/09593985.2018.1551455, 30501424

[15] Posner M Rothbart M. Attention, self-regulation and consciousness. Philos Trans R Soc Lond Ser B Biol Sci. (1998) 353:1915–27. doi: 10.1098/rstb.1998.0344, 9854264 PMC1692414

[16] Olivier I Cuisinier R Vaugoyeau M Nougier V Assaiante C. Age-related differences in cognitive and postural dual-task performance. Gait Posture. (2010) 32:494–9. doi: 10.1016/j.gaitpost.2010.07.008, 20692161

[17] Palluel E Nougier V Olivier I. Postural control and attentional demand during adolescence. Brain Res. (2010) 1358:151–9. doi: 10.1016/j.brainres.2010.08.051, 20735993

[18] Klotzbier TJ Bühler K Holfelder B Schott N. Exploring motor-cognitive interference in children with Down syndrome using the trail-walking-test. Res Dev Disabil. (2020) 106:103769. doi: 10.1016/j.ridd.2020.103769, 32979845

[19] Schott N. Dual-task performance in developmental coordination disorder (DCD): understanding trade-offs and their implications for training. Curr Dev Disord Rep. (2019) 6:87–101. doi: 10.1007/s40474-019-00163-z

[20] Vandoni M Giuriato M Pirazzi A Zanelli S Gaboardi F Carnevale Pellino V . Motor skills and executive functions in pediatric patients with Down syndrome: a challenge for tailoring physical activity interventions. Pediatr Rep. (2023) 15:691–706. doi: 10.3390/pediatric15040062, 37987287 PMC10661287

[21] Lanfranchi S Baddeley A Gathercole S Vianello R. Working memory in Down syndrome: is there a dual task deficit? J Intellect Disabil Res. (2012) 56:157–66. doi: 10.1111/j.1365-2788.2011.01444.x, 21726323

[22] Malak R Kostiukow A Krawczyk-Wasielewska A Mojs E Samborski W. Delays in motor development in children with Down syndrome. Med Sci Monit. (2015) 21:1904–10. doi: 10.12659/msm.893377, 26132100 PMC4500597

[23] Austin H Balendra N Langenderfer J Ustinova K. Decomposition of leg movements during overground walking in individuals with traumatic brain injury. Brain Inj. (2018) 32:739–46. doi: 10.1080/02699052.2018.1444203, 29494269

[24] Leach JM Mancini M Kaye JA Hayes TL Horak FB. Day-to-day variability of postural sway and its association with cognitive function in older adults: a pilot study. Front Aging Neurosci. (2018) 10:126. doi: 10.3389/fnagi.2018.00126, 29780319 PMC5945980

[25] Liu Y-C Yang Y-R Tsai Y-A Wang R-Y. Cognitive and motor dual task gait training improve dual task gait performance after stroke—a randomized controlled pilot trial. Sci Rep. (2017) 7:4070. doi: 10.1038/s41598-017-04165-y, 28642466 PMC5481328

[26] Stegemöller EL Wilson JP Hazamy A Shelley MC Okun MS Altmann LJ . Associations between cognitive and gait performance during single- and dual-task walking in people with Parkinson’s disease. Phys Ther. (2014) 94:757–66. doi: 10.2522/ptj.20130251, 24557652 PMC4040423

[27] Woollacott M Shumway-Cook A. Attention and the control of posture and gait: a review of an emerging area of research. Gait Posture. (2002) 16:1–14. doi: 10.1016/s0966-6362(01)00156-4, 12127181

[28] Ko S-u Jerome GJ Simonsick EM Studenski S Hausdorff JM. Differential associations between dual-task walking abilities and usual gait patterns in healthy older adults—results from the Baltimore longitudinal study of aging. Gait Posture. (2018) 63:63–7. doi: 10.1016/j.gaitpost.2018.04.039, 29723649 PMC6106773

[29] Pena GM Pavão SL Oliveira MFP Godoi D de Campos AC Rocha N. Dual-task effects on postural sway during sit-to-stand movement in children with Down syndrome. J Intellect Disabil Res. (2019) 63:576–86. doi: 10.1111/jir.12599, 30687997

[30] Büyükçelik NM Yiğit S Turhan B. An investigation of the effects of dual-task balance exercises on balance, functional status, and dual-task performance in children with Down syndrome. Dev Neurorehabil. (2023) 26:320–7. doi: 10.1080/17518423.2023.2233031, 37403442

[31] Horvat M Croce R Tomporowski P. The influence of dual-task conditions on movement in young adults with and without Down syndrome. Res Dev Disabil. (2013) 34:3517–25. doi: 10.1016/j.ridd.2013.06.038, 23962599

[32] Borji R Laatar R Zarrouk N Sahli S Rebai H. Cognitive-motor interference during standing stance across different postural and cognitive tasks in individuals with Down syndrome. Res Dev Disabil. (2023) 139:104562. doi: 10.1016/j.ridd.2023.104562, 37379660

[33] Hocking DR Menant JC Kirk HE Lord S Porter MA. Gait profiles as indicators of domain-specific impairments in executive control across neurodevelopmental disorders. Res Dev Disabil. (2014) 35:203–14. doi: 10.1016/j.ridd.2013.10.005, 24176260

[34] Page MJ McKenzie JE Bossuyt PM Boutron I Hoffmann TC Mulrow CD . The PRISMA 2020 statement: an updated guideline for reporting systematic reviews. Int J Surg. (2021) 88:105906. doi: 10.1016/j.ijsu.2021.105906, 33789826

[35] Sterne JA Hernán MA Reeves BC Savović J Berkman ND Viswanathan M . ROBINS-I: a tool for assessing risk of bias in non-randomised studies of interventions. BMJ. (2016) 355:i4919. doi: 10.1136/bmj.i4919 27733354 PMC5062054

[36] Sterne JA Savović J Page MJ Elbers RG Blencowe NS Boutron I . RoB 2: a revised tool for assessing risk of bias in randomised trials. BMJ. (2019) 366:l4898. doi: 10.1136/bmj.l4898 31462531

[37] Campbell M McKenzie JE Sowden A Katikireddi SV Brennan SE Ellis S . Synthesis Without Meta-Analysis (SWiM) in systematic reviews: reporting guideline. BMJ. (2020) 368:l6890. doi: 10.1136/bmj.l6890 31948937 PMC7190266

[38] Triki A Borji R Laatar R Sahli S Rebai H. The effect of dual-task training on postural and cognitive performances in adolescents with Down syndrome. Res Dev Disabil. (2024) 153:104827. doi: 10.1016/j.ridd.2024.104827, 39216176

[39] Uysal F Belgen Kaygisiz B Cavlak U. The effect of dual task on walking speed and cadence in children with Down syndrome. Arch Health Sci Res. (2021) 8:33–8. doi: 10.5152/ArcHealthSciRes.2021.20090

[40] Leone C Feys P Moumdjian L D’Amico E Zappia M Patti F. Cognitive-motor dual-task interference: a systematic review of neural correlates. Neurosci Biobehav Rev. (2017) 75:348–60. doi: 10.1016/j.neubiorev.2017.01.010, 28104413

[41] Haith AM Krakauer JW. The multiple effects of practice: skill, habit and reduced cognitive load. Curr Opin Behav Sci. (2018) 20:196–201. doi: 10.1016/j.cobeha.2018.01.015, 30944847 PMC6443249

[42] Baladaniya M Baldania S Hait A Choudhary AK Hait A. Dual-task gait and balance training integrated with sensory-motor interventions for children with autism spectrum disorder: a comprehensive narrative review. Cureus. (2025) 17:e93268. doi: 10.7759/cureus.93268, 41146756 PMC12554153

[43] Jain PD Nayak A Karnad SD Doctor KN. Gross motor dysfunction and balance impairments in children and adolescents with Down syndrome: a systematic review. Clin Exp Pediatr. (2022) 65:142–9. doi: 10.3345/cep.2021.00479, 34126707 PMC8898616

[44] Beerse M Henderson G Liang H Ajisafe T Wu J. Variability of spatiotemporal gait parameters in children with and without Down syndrome during treadmill walking. Gait Posture. (2019) 68:207–12. doi: 10.1016/j.gaitpost.2018.11.032, 30504087

[45] Travers BG Mason A Gruben KG Dean DC 3rd McLaughlin K. Standing balance on unsteady surfaces in children on the autism spectrum: the effects of IQ. Res Autism Spectr Disord. (2018) 51:9–17. doi: 10.1016/j.rasd.2018.03.008, 30333859 PMC6186444

