# Supplementary material for: The effect of dual-task on postural control and gait in individuals with Down syndrome: a systematic review
Source: Front Neurol. 2026 Jun 11;17:1858461. doi: 10.3389/fneur.2026.1858461 (PMC13295179; doi:10.3389/fneur.2026.1858461)
Supplement: Supplementary file 1 [file Table_1.DOCX]

**Supplementary Table 1.** Databases search strategies.

| **Database** | **Complete search strategy** | **Documents retrieved** |
| --- | --- | --- |
| **Web of Science** | TS = ("dual task*" OR "divided attention" OR "concurrent task*" OR "cognitive task*" OR "multi task*" OR "double task*" OR "motor task*" OR "cognitive-motor interference" OR "secondary task" OR "walking while talking") AND TS = (posture OR "postural control" OR "postural sway" OR "postural stability" OR "postural steadiness" OR balance OR equilibrium OR "postural balance" OR gait OR walk* OR tread* OR step* OR locomotion OR mobility OR ambulation) AND TS = ("down syndrome" OR "down's syndrome" OR "21 trisomy" OR "trisomy 21") | 21 |
| **PubMed** | (("Dual Task"[Mesh] OR "dual task"[tiab] OR "dual-task*"[tiab] OR "divided attention"[tiab] OR "concurrent task"[tiab] OR "cognitive task"[tiab] OR "multi task"[tiab] OR "double task"[tiab] OR "motor task"[tiab] OR "cognitive-motor interference"[tiab] OR "secondary task"[tiab] OR "walking while talking"[tiab]) AND ("Postural Balance"[Mesh] OR posture[tiab] OR "postural control"[tiab] OR "postural sway"[tiab] OR "postural stability"[tiab] OR "postural steadiness"[tiab] OR balance[tiab] OR equilibrium[tiab] OR "postural balance"[tiab] OR gait[tiab] OR walk[tiab] OR tread[tiab] OR step[tiab] OR locomotion[tiab] OR mobility[tiab] OR ambulation[tiab])) AND ("Down Syndrome"[Mesh] OR "down syndrome"[tiab] OR "down's syndrome"[tiab] OR "21 trisomy"[tiab] OR "trisomy 21"[tiab]) | 13 |
| **Scopus** | TITLE-ABS-KEY ("dual task*" OR "divided attention" OR "concurrent task*" OR "cognitive task*" OR "multi task*" OR "double task*" OR "motor task*" OR "cognitive-motor interference" OR "secondary task" OR "walking while talking") AND TITLE-ABS-KEY (posture OR "postural control" OR "postural sway" OR "postural stability" OR "postural steadiness" OR balance OR equilibrium OR "postural balance" OR gait OR walk* OR tread* OR step* OR locomotion OR mobility OR ambulation) AND TITLE-ABS-KEY ("down syndrome" OR "down's syndrome" OR "21 trisomy" OR "trisomy 21") | 27 |

**Supplementary Table 2.** Excluded studies after assessing for eligibility.

| **Population** | **Exclusion reason** |
| --- | --- |
| 1. Olivier, I., Cuisinier, R., Vaugoyeau, M., Nougier, V., & Assaiante, C. (2010). Age-related differences in cognitive and postural dual-task performance. Gait & posture, 32(4), 494–499. | Individuals without Down syndrome |
| 2. Liu, Y. C., Yang, Y. R., Tsai, Y. A., & Wang, R. Y. (2017). Cognitive and motor dual task gait training improve dual task gait performance after stroke - A randomized controlled pilot trial. Scientific reports, 7(1), 4070. | Individuals with Stroke |
| 3. Stegemöller, E. L., Wilson, J. P., Hazamy, A., Shelley, M. C., Okun, M. S., Altmann, L. J., & Hass, C. J. (2014). Associations between cognitive and gait performance during single- and dual-task walking in people with Parkinson disease. Physical therapy, 94(6), 757–766. | Individuals with Parkinson |
| 4. Skaletski EC, Cardona SC, Travers BG. The relation between specific motor skills and daily living skills in autistic children and adolescents. Front Integr Neurosci. 2024;18. | Individuals on the autism spectrum |
| **Intervention** | |
| 1.Naito, M., Aoki, S., Kamide, A., Miyamura, K., Honda, M., Nagai, A., Mezawa, H.,and Hashimoto, K. (2015) Gait analysis in Down syndrome pediatric patients using a sheet-type gait analyzer: Pilot study. *Pediatrics International*, 57: 860–863. | Absence of dual task |
| 2. Kokubun M. Are children with down syndrome less careful in performing a tray-carrying task than children with other types of mental retardation? Percept Mot Skills. 1999;88(3 PART 2):1173-6. | Absence of dual task |
| 3. Chen HL, Yeh CF, Howe TH. Postural control during standing reach in children with Down syndrome. Res Dev Disabil. 2015;38:345-51. | Absence of dual task |
| **Outcome** | |
| 1. Lanfranchi, S., Baddeley, A., Gathercole, S., & Vianello, R. (2012). Working memory in Down syndrome: is there a dual task deficit?. *Journal of intellectual disability research : JIDR*, *56*(2), 157–166. | Absence of postural control variables |
| 2. Van Pelt, K. L., Koehl, L., Caban-Holt, A., Anderson-Mooney, A., Head, E., & Schmitt, F. A. (2020). Feasibility of dual-task gait to estimate Alzheimer's related cognitive decline in Down syndrome. *Alzheimer's & dementia (Amsterdam, Netherlands)*, *12*(1), e12092. | Absence of postural control variables |
| 3. Holfelder, B., Klotzbier, T. J., & Schott, N. (2022). Dual-Task Interference in Children with Down Syndrome and Chronological and Mental Age-Matched Healthy Controls. *Children*, *9*(2), 191. | Absence of postural control variables |
| 4. Vimercati SL, Galli M, Stella G, Caiazzo G, Ancillao A, Albertini G. Clumsiness in fine motor tasks: Evidence from the quantitative drawing evaluation of children with Down Syndrome. J Intell Disabil Res. 2015;59(3):248-56. | Absence of postural control variables |
| **Design** | |
| 1. Smith, B. A., Stergiou, N., & Ulrich, B. D. (2011). Patterns of gait variability across the lifespan in persons with and without Down syndrome. *Journal of Neurologic Physical Therapy, 35*(4), 170–177. | Cross-sectional study |
| 2. Malak, R., Kostiukow, A., Krawczyk-Wasielewska, A., Mojs, E., & Samborski, W. (2015). Delays in Motor Development in Children with Down Syndrome. *Medical science monitor : international medical journal of experimental and clinical research*, *21*, 1904–1910. | Comparative study |
